# Presence of rare potential pathogenic variants in subjects under 65 years old with very severe or fatal COVID-19

**DOI:** 10.1038/s41598-022-14035-x

**Published:** 2022-06-20

**Authors:** Rosario López-Rodríguez, Marta Del Pozo-Valero, Marta Corton, Pablo Minguez, Javier Ruiz-Hornillos, María Elena Pérez-Tomás, María Barreda-Sánchez, Esther Mancebo, Cristina Villaverde, Gonzalo Núñez-Moreno, Raquel Romero, The STOP_Coronavirus Study Group, Lidia Fernández-Caballero, Ruth Fernández Sanchez, Inés García Vara, Laura Marzal Gordo, Andrea Martinez-Ramas, Lorena Ondo, Miguel Górgolas, Alfonso Cabello, Germán Peces Barba, Sara Heili, César Calvo, María Dolores Martín Ríos, Arnoldo Santos, Olga Sánchez-Pernaute, Lucía Llanos, Sandra Zazo, Federico Rojo, Felipe Villar, Raimundo de Andrés, Ignacio Jiménez Alfaro, Ignacio Gadea, Celia Perales, Yolanda Cañadas Juarez, Ignacio Mahillo, Antonio Herrero, Juan Carlos Taracido, Elisa García-Vázquez, Rubén Jara-Rubio, José A. Pons-Miñano, Juana M. Marín-Martínez, M. Teresa Herranz-Marín, Enrique Bernal-Morell, Josefina García-García, Juan de Dios González-Caballero, M. Dolores Chirlaque-López, Alfredo Minguela-Puras, Manuel Muro-Amador, Antonio Moreno-Docón, Genoveva Yagüe-Guirao, José M. Abellán-Perpiñán, Jorge E. Martínez-Pérez, Fernando I. Sánchez-Martínez, Alberto Utrero-Rico, Mario Fernández-Ruiz, Octavio Carretero, José María Aguado, Rocio Laguna-Goya, Ángel Jiménez, María Herrera Abián, Mercedes García Salmones, Lidia Gagliardi Alarcon, María Rubio Oliveira, Carlos Fabian Castaño Romero, Carlos Aranda Cosgaya, Virginia Víctor Palomares, Leticia García Rodríguez, Maria Sanchez Carpintero Abad, Mª Carmen García Torrejón, Estela Paz-Artal, Encarna Guillén-Navarro, Berta Almoguera, Carmen Ayuso

**Affiliations:** 1 Department of Genetics & Genomics, Instituto de Investigación Sanitaria-Fundación Jiménez Díaz University Hospital, Universidad Autónoma de Madrid (IIS-FJD, UAM), Madrid, Spain; 2 Center for Biomedical Network Research on Rare Diseases (CIBERER), Instituto de Salud Carlos III, 28029 Madrid, Spain; 3 Bioinformatics Unit, Instituto de Investigación Sanitaria-Fundación Jiménez Díaz University Hospital, Universidad Autónoma de Madrid (IIS-FJD, UAM), Madrid, Spain; 4 Allergy Unit, Hospital Infanta Elena, Valdemoro, Madrid, Spain; 5 Instituto de Investigación Sanitaria-Fundación Jiménez Díaz University Hospital, Universidad Autónoma de Madrid (IIS-FJD, UAM), Madrid, Spain; 6 Faculty of Medicine, Universidad Francisco de Vitoria, Pozuelo de Alarcón, Madrid, Spain; 7 Instituto Murciano de Investigación Biosanitaria Virgen de la Arrixaca (IMIB-Arrixaca), Murcia, Spain; 8 Health Sciences Faculty, Universidad Católica San Antonio de Murcia (UCAM), Murcia, Spain; 9 Department of Immunology, Hospital Universitario 12 de Octubre, Madrid, Spain; 10 Instituto de Investigación Sanitaria Hospital 12 de Octubre (imas12), Madrid, Spain; 11 Department of Immunology, Ophthalmology and ENT, Universidad Complutense de Madrid, Madrid, Spain; 12 Center for Biomedical Network Research on Infectious Diseases (CIBERINFEC), Instituto de Salud Carlos III, 28029 Madrid, Spain; 13 Medical Genetics Section, Pediatric Department, Virgen de la Arrixaca University Clinical Hospital, Faculty of Medicine, University of Murcia (UMU), Murcia, Spain; 14 Present Address: Department of Pharmaceutical and Health Sciences, Faculty of Pharmacy, Universidad San Pablo-CEU, CEU Universities, Madrid, Spain; 15 Division of Infectious Diseases, Instituto de Investigación Sanitaria-Fundación Jiménez Díaz University Hospital, Universidad Autónoma de Madrid (IIS-FJD, UAM), Madrid, Spain; 16 Department of Neumology, Instituto de Investigación Sanitaria-Fundación Jiménez Díaz University Hospital, Universidad Autónoma de Madrid (IIS-FJD, UAM), Madrid, Spain; 17 Intensive Care Department, Instituto de Investigación Sanitaria-Fundación Jiménez Díaz University Hospital, Universidad Autónoma de Madrid (IIS-FJD, UAM), Madrid, Spain; 18 Preventive Medicine Department, Instituto de Investigación Sanitaria-Fundación Jiménez Díaz University Hospital, Universidad Autónoma de Madrid (IIS-FJD, UAM), Madrid, Spain; 19 Reumathology Service, Instituto de Investigación Sanitaria-Fundación Jiménez Díaz University Hospital, Universidad Autónoma de Madrid (IIS-FJD, UAM), Madrid, Spain; 20 Clinical Trials Unit, Instituto de Investigación Sanitaria-Fundación Jiménez Díaz University Hospital, Universidad Autónoma de Madrid (IIS-FJD, UAM), Madrid, Spain; 21 Department of Pathology, Instituto de Investigación Sanitaria-Fundación Jiménez Díaz University Hospital, Universidad Autónoma de Madrid (IIS-FJD, UAM), BiobankMadrid, Spain; 22 Internal Medicine Department, Instituto de Investigación Sanitaria-Fundación Jiménez Díaz University Hospital, Universidad Autónoma de Madrid (IIS-FJD, UAM), Madrid, Spain; 23 Opthalmology Department, Instituto de Investigación Sanitaria-Fundación Jiménez Díaz University Hospital, Universidad Autónoma de Madrid (IIS-FJD, UAM), Madrid, Spain; 24 Department of Clinical Microbiology, Instituto de Investigación Sanitaria-Fundación Jiménez Díaz University Hospital, Universidad Autónoma de Madrid (IIS-FJD, UAM), Av. Reyes Católicos 2, 28040 Madrid, Spain; 25 Instituto de Investigación Sanitaria-Fundación Jiménez Díaz University Hospital, Universidad Autónoma de Madrid (IIS-FJD, UAM), Madrid, Spain; 26 Department of Statistics, Instituto de Investigación Sanitaria-Fundación Jiménez Díaz University Hospital, Universidad Autónoma de Madrid (IIS-FJD, UAM), Av. Reyes Católicos 2, 28040 Madrid, Spain; 27 Data Analysis Department, Instituto de Investigación Sanitaria-Fundación Jiménez Díaz University Hospital, Universidad Autónoma de Madrid (IIS-FJD, UAM), Av. Reyes Católicos 2, 28040 Madrid, Spain; 28 Hospital Clínico Universitario Virgen de la Arrixaca, Murcia, Spain; 29 Servicio de Medicina Intensiva, Hospital Clínico Universitario Virgen de la Arrixaca, Murcia, Spain; 30 Servicio de Digestivo, Hospital Clínico Universitario Virgen de la Arrixaca, Murcia, Spain; 31 Servicio de Urgencias, Hospital Clínico Universitario Virgen de la Arrixaca, Murcia, Spain; 32 Servicio de Medicina Interna, Hospital Universitario Morales Meseguer, Murcia, Spain; 33 Unidad de Enfermedades Infecciosas, Hospital General Universitario Reina Sofía, Murcia, Spain; 34 Servicio de Medicina Interna, Hospital Universitario Santa Lucía, Murcia, Spain; 35 Programa deCuidados y Cronicidad del Sistema Murciano de Salud, Subdirección General de Planificación, Murcia, Spain; 36 Servicio de Epidemiología de la Consejería de Salud, Murcia, Spain; 37 Servicio de Inmunología, Hospital Clínico Universitario Virgen de la Arrixaca, Murcia, Spain; 38 Servicio de Microbiología, Hospital Clínico Universitario Virgen Arrixaca, Murcia, Spain; 39 Departamento de Economía Aplicada, Universidad de Murcia, Murcia, Spain; 40 Unit of Infectious Diseases, Hospital Universitario “12 de Octubre”, Instituto de Investigación Sanitaria Hospital “12 de Octubre” (imas12), Madrid, Spain; 41 Department of Medicine, School of Medicine, Universidad Complutense, Madrid, Spain; 42 Department of Internal Medicine, Infanta Elena University Hospital, Valdemoro, Madrid, Spain; 43 Faculty of Medicine, Universidad Francisco de Vitoria, Pozuelo de Alarcón, Madrid, Spain; 44 Department of Geriatrics & Paliative Care, Infanta Elena University Hospital, Valdemoro, Madrid, Spain; 45 Department of Pneumology, Infanta Elena University Hospital, Valdemoro, Madrid, Spain; 46 Intensive Care Unit, Infanta Elena University Hospital, Valdemoro, Madrid, Spain

**Keywords:** Clinical genetics, Genetics research

## Abstract

Rare variants affecting host defense against pathogens could be involved in COVID-19 severity and may help explain fatal outcomes in young and middle-aged patients. Our aim was to report the presence of rare genetic variants in certain genes, by using whole exome sequencing, in a selected group of COVID-19 patients under 65 years who required intubation or resulting in death (n = 44). To this end, different etiopathogenic mechanisms were explored using gene prioritization-based analysis in which genes involved in immune response, immunodeficiencies or blood coagulation were studied. We detected 44 different variants of interest, in 29 different patients (66%). Some of these variants were previously described as pathogenic and were located in genes mainly involved in immune response. A network analysis, including the 42 genes with candidate variants, showed three main components, consisting of 25 highly interconnected genes related to immune response and two additional networks composed by genes enriched in carbohydrate metabolism and in DNA metabolism and repair processes. In conclusion, we have detected candidate variants that may potentially influence COVID-19 outcome in our cohort of patients. Further studies are needed to confirm the ultimate role of the genetic variants described in the present study on COVID-19 severity.

## Introduction

Since the first outbreak of the Coronavirus disease in the 2019 (COVID-19) pandemic, over 243 million cases of COVID-19 and more than 6.1 million deaths have been confirmed (https://coronavirus.jhu.edu, *last accessed* in March 2022). SARS-CoV-2 infection displays high inter-individual clinical variability, ranging from asymptomatic to lethal outcomes^1^. The most important life-threatening factor is age, increasing the risk for critical illness for individuals over 65 years of age^2^. Other known risk factors are being male and having comorbidities such as hypertension, diabetes and cardiovascular, renal or respiratory diseases^3,4^. However, these risk factors do not explain completely why apparently healthy young and middle-aged adults present severe COVID-19 with acute respiratory distress syndrome (ARDS) that cause a fulminant disease in some cases.

Genetic background has been proposed as a candidate factor to explain some of the inter-individual variability observed in COVID-19 severity. Recently, different genome-wide association studies (GWAS) have identified several loci associated with an increased susceptibility to SARS-CoV-2 infection and severe disease^5–7^.These loci include genes involved in type I interferon (IFN) signaling pathway (*IFNAR2, DPP9 or OAS1-3*), autoimmunity (*TYK2*) or in lung function (*FOXP4*).However, top associated variants displayed low odd ratios to be considered predictive biomarkers of COVID-19 severity^5–7^.

In addition to common variants detected in GWAS, rare variants affecting host defense against pathogens could be involved in COVID-19 severity and may help explain fatal outcomes in young and middle-aged patients. In fact, inborn errors of immunity producing increased infection susceptibility and/or infection recurrence (such as primary immunodeficiencies or N-glycosylation defects), may aggravate the course of SARS-CoV-2 infection^8–10^. Enrichment in loss-of-function (LoF) variants in 13 genes belonging to type I IFN signaling pathway has been reported in patients with life-threatening COVID-19 pneumonia^11^, although this finding has not been replicated^12^. Moreover, LoF genetic variants in Toll-like receptor 7 (*TLR7*), which is critical in the recognition of single-stranded RNA viruses and fostering the antiviral responses, have been associated to more severe outcomes in young males without comorbidities^13–16^.

Apart from ARDS, thrombosis and coagulopathy emerge as critical complications of SARS-CoV-2 infection^17–19^. In fact, elevation of the thrombotic related D-dimer is one of the most frequent laboratory findings, particularly in critically ill patients^20^. In addition, different studies have shown the influence of ABO blood groups on the risk of SARS-CoV-2 infection and/or the severity of the disease^21^. In this regard, the demonstrated impact of ABO phenotypes on vascular homeostasis and function^22^, has been suggested as an explanation of the reported associations between COVID-19 severity and ABO blood groups. Thus, pathogenic genetic variants altering protein functionality of coagulation system may also impact on COVID-19 resolution.

Based on this evidence, our objective was to identify rare genetic variants related to COVID-19 severity. To this end, we selected a group of patients under 65 years who experienced a very severe outcome defined as requiring intubation or resulting in death and were subjected to whole exome sequencing. Different etiopathogenic mechanisms were explored using gene prioritization-based analysis in which more than 800 genes involved in immune response, immunodeficiencies or blood coagulation were studied.

## Results

### Clinical and demographic characteristics

A total of 44 unrelated patients with very severe COVID-19) that required intubation, non-invasive ventilatory support or did not survive to SARS-CoV-2 infection were included in the present study. A total of 16 patients (36%) did not survive, most of them received invasive ventilatory support (intubation, 44%) and 31% received exclusively non-invasive ventilatory support (Table 1). The median age was 46 years, and patients were mainly males (70%, Table 1). Most patients were of European ancestry (86%), except for 7 Admixed Americans and one patient from another ethnicity. Main clinical characteristics of this cohort such as pre-existing diseases and COVID-19 management are summarized in Table 1. The most frequent comorbidities were obesity (defined as body mass index > 33 kg/m^2^; 39%), hypertension (18%), respiratory disease (16%) and oncohematological antecedents (14%). Detailed clinical data of each patient are provided in Supplementary Table 1.

**Table 1:** Clinical and demographic characteristics.

	All (n = 44)	Survivors (n = 28)	Deceased (n = 16)
Males (n, %)	31, 70%	23, 82%	8, 50%
Age (median, range)	46, 24–62	42, 24–50	56, 45–62
Europeans (n, %)	36, 82%	20, 71%	16, 100%
**Pre-existing conditions (n, %)**			
Hypertension	8, 18%	5, 18%	3, 19%
Hypercholesterolemia	3, 7%	1, 4%	2, 13%
Type 2 diabetes	5, 11%	3, 11%	2, 13%
Obesity	17, 39%	9, 32%	8, 50%
Cardiovascular disease	4, 9%	2, 7%	2, 13%
Neurological disease	2, 4.5%	1, 4%	1, 6%
Respiratory disease	7, 16%	3, 11%	4, 25%
Digestive/liver disease	4, 9%	3, 11%	1, 6%
Oncohematological antecedents	6, 14%	1, 4%	5, 31%
Kidney disease	2, 4.5%	0	2, 13%
**Management of COVID-19 disease**			
ICU (n, %)	39, 89%	28, 100%	11, 69%
Invasive ventilation (intubation, n, %)	35, 80%	28, 100%	7, 44%
Non-invasive ventilation (CPAP/BiPAP/high flow nasal cannula)^a^ (n, %)	5, 11%%	0, 0%	5, 31%%
Without ventilatory support	4, 9%	0, 0%	4, 25%
Hospitalization (mean ± SD of days)	33.2 ± 24.8	38.8 ± 26.3	24.0 ± 19.3

^a^Patients that received exclusively non-invasive ventilatory support.

### Identification of candidate variants

**Figure 1:**
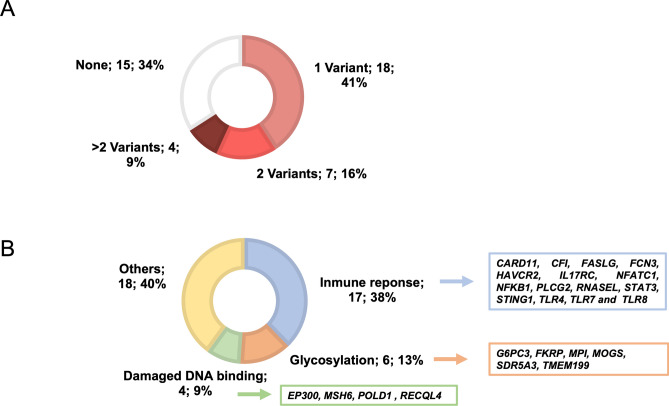
Identification of variants of interest in very severe COVID-19 patients: frequency and functional pathways involved. (**A**) Number and percentage of patients with none, one, two or more variants of interest, (**B**) Number and percentage of variants detected in each of the functional pathways.

We have detected 44 different variants of interest located in 42 genes. These variants were identified in 29 patients, 11 of them carrying 2 or more candidate variants in different genes (Fig. 1A). Available data about pathogenic predictors, MAF and pathogenic annotation from public databases (*ClinVar* and *HGMD*) are summarized in Supplementary Table 2. A total of 12 (26%) variants were previously described as likely pathogenic or displayed strong evidence for being considered as likely pathogenic following ACMG criteria (Table 2).

**Table 2:** List of filtered candidate variants identified in our cohort of COVID-19 patients.

Patient	Age	Sex	O	Gene	IM	GT	HGVSc	HGVSp	Varsome Class^a^	LP/P
FJD_0004	46	M	S	*PLAU*	AD	HT	NM_002658.3:c.970 + 1G>A	p.?	LP	No
FJD_0019	50	M	S	*NPHS1*	AR	HT	NM_004646.3:c.767G>A	p.Arg256Gln	LP	No
FJD_0044	49	M	S	G6PD	X-L	HM	NM_000402.4:c.934G>C	p.Asp312His	LP	Yes^b^
				*NFATC1*	AD	HT	NM_006162.5: c.230C>T	p.Pro77Leu	VUS	No
				*TLR8*	X-L	HM	NM_016610.3:c.2263A>T	p.Ser755Cys	VUS	No
FJD_0081	24	M	S	*CARD11*	AD	HT	NM_032415.7:c.572A>G	p.Asn191Ser	VUS	No
FJD_0325	57	M	S	*PDGFRA*	AD	HT	NM_006206.6:c.2464C>T	p.Arg847Cys	VUS	No
				*SLC9A3*	AR	HT	NM_004174.2:c.1144C>T	p.Arg382Trp	LP	No
FJD_0374	58	M	D	*FANCD2*	AR	HT	NM_033084.3:c.2444G>A	p.Arg815Gln	LP	Yes^b^
				*FCN3*	AR	HT	NM_003665.2:c.232 + 1G>A	p.?	LP	No^b^
				*FKRP*	AR	HT	NM_024301.4:c.265C>T	p.Pro89Ser	LP	No
				*MYO5B*	AR	HT	NM_001080467.2:c.3843G>C	p.Ala1281Ala	LP	No
FJD_0412	60	F	D	*POLR3C*	AD	HT	NM_001303456.1:c.706G>C	p.Asp236His	VUS	No
FJD_0626	59	F	D	*FN1*	AD	HT	NM_212482.2:c.4654C>G	p.Pro1552Ala	VUS	No
				*MOGS*	AR	HT	NM_006302.2:c.882del	p.Glu295Asnfs*10	LP	No^b^
				*POLD1*	AR	HT	NM_002691.4:c.378_394del	p.Ala127*	LP	No
FJD_0714	44	M	S	*IL17RC*	AR	HT	NM_153461.3:c.1132C>T	p.Arg378*	LP	No^b^
FJD_0728	62	F	D	*SRD5A3*	AR	HT	NM_024592.4:c.57G>A	p.Trp19*	LP	Yes^b^
				*STAT3*	AD	HT	NM_139276.3:c.523A>C	p.Asn270His	VUS	No
FJD_1417	38	M	S	*PLCG2*	AD	HT	NM_002661.4:c.3022C>T	p.Gln1008*	LP	No
FJD_1456	49	F	D	*G6PC3*	AR	HT	NM_138387.3:c.889del	p.Leu297Trpfs*27	LP	No^b^
				*MPI*	AR	HT	NM_002435.1:c.1123G>C	p.Gly375Arg	LP	No
				*PIK3CD*	AD	HT	NM_005026.5:c.2869C>T	p.Arg981Trp	VUS	No
FJD_1458	56	M	D	*NFATC1*	AD	HT	NM_006162.5:c.2311C>A	p.Leu771Ile	VUS	No
FJD_1462	51	F	D	*IL17RC*	AR	HT	NM_153461.3:c.1132C>T	p.Arg378 *	LP	No^b^
FJD_1532	46	M	S	*TMEM199*	AR	HT	NM_152464.1:c.92G>C	p.Arg31Pro	LP	Yes^b^
FJD_1645	59	M	D	*RECQL4*	AR	HT	NM_004260.3:c.2756G>A	:p.Ala919Thr	LP	No
FJD_2149	53	M	D	*MSH6*	AR	HT	NM_000179.2:c.3226C>T	p.Arg1076Cys	LP	Yes^b^
H12O_104	30	M	S	*TLR7*	X-L	HM	NM_016562.3:c.2050A>T	p.Lys684*	LP	Yes^b^
H12O_107	38	F	S	*EP300*	AD	HT	NM_001429.3:c.7180G>C	p.Gly2394Arg	VUS	No
				*SLC37A4*	AR	HT	NM_001164277.1:c.1016G>T	p.Gly339Cys	LP	Yes^b^
H12O_110	27	M	S	*POLR3C*	AD	HT	NM_001303456.1:c.1597A>T	p.Ile533Phe	VUS	No
H12O_111	45	F	D	*NFKB1*	AD	HT	NM_003998.4:c.233A>G	p.Asn86Ser	VUS	No
H12O_112	42	M	S	*CFI*	AR	HT	NM_000204.5:c.1643A>G	p.Glu556Gly	LP	No
				*LDLR*	AD	HT	NM_000527.4:c.1816G>A	p.Ala606Thr	LP	No
H12O_113	45	M	S	*RNASEL*	AD	HT	NM_021133.3:c.1450C>T	p.Gln484*	VUS	No
H12O_236	53	M	D	*FASLG*	AD	HT	NM_000639.1:c.596G>T	p.Gly199Val	VUS	No
				*HAVCR2*	AR	HT	NM_032782.4:c.291A>G	p.Ile97Met	LP	No
HVAM_083	48	M	S	*SLX4*	AR	HT	NM_032444.2:c.2340_2343del	p.Glu781Serfs*38	LP	No^b^
				*TLR4*	AD	HT	NM_138554.4:c.1976T>C	p.Met659Thr	VUS	No
HVAM_137	41	M	S	*PTPN11*	AD	HT	NM_002834.3:c.369G>T	p.Glu123Asp	VUS	No
HVAM_142	49	M	S	*STING1*	AD	HT	NM_198282.2:c.65C>A	p.Ala22Asp	VUS	No
HVAM_212	46	M	S	*CHD7*	AD	HT	NM_017780.3:c.8257A>G	p.Met2753Val	VUS	No
				*TEK*	AD	HT	NM_000459.3:c.2357A>G	p.Gln786Arg	VUS	No
HVAM_252	44	M	S	*ITGB3*	AD	HT	NM_000212.2:c.1658_1660del	p.Ser553del	VUS	No

*M* male, *F* female, *O* Outcome, *D* deceased, *S* survivor, *IM* inheritance mode, *AD* autosomal dominant, *AR* autosomal recessive, *X-L* linked to X chromosome, *GT* genotype, *HT* heterozygous, *HM* Hemizygous, *Chr *chromosome, *HGVSc* HGVS coding sequence name, *HGVSp* HGVS protein sequence name, *LP* likely pathogenic, *P* pathogenic, *VUS* variant of unknown significance. ^a^*Varsome* class followingACMG criteria; ^b^variants previously described as likely pathogenic or displaying strong evidence for being considered as likely pathogenic.

Thirty eight percent of the identified variants (n = 17, 15 different variants and 1 variant found in 2 cases) was located in 15 genes involved in immune response (Fig. 1B). Eight of these candidate variants were found in genes with an autosomal dominant inheritance mode (such as *PLCG2*, *RNASEL*, *TLR4* or* STAT3*), six variants were detected in genes with an autosomal recessive inheritance mode (*CFI*, *FCN3*, *HAVCR2* and *IL17RC*) and three variants were identified in X-linked genes (*TLR7*, *TLR8* and *G6PD*). The detected LoF variant in *TLR7* was found in a 30-year-old male who was included in a case-series recently reported^16^. The p.Arg378* variant on *IL17RC*, which was not described in public databases (*GnomAD*^23^*, ExAc*^24^ or 1000 genome project^25^), was detected in two unrelated patients from our cohort (MAF of 2.3%). The patient carrying the variant p.Ser755Cys located in the *TLR8* gene was also hemizygous for a pathogenic variant in *G6PD* (both XL genes) and did not show pre-existing comorbidities or risk factors at the time of the SARS-CoV-2 infection (Supplementary Table 1). Besides, one of the deceased patients, clinically diagnosed with a primary immunodeficiency (FJD_0728); carried a variant on the *STAT3* gene (p.Asn175His), in addition to one pathogenic variant in the recessive *SRD5A3* gene (Table 2).

A total of 6 candidate variants (14%) were found in genes related to congenital disorders of glycosylation (*G6PC3*, *FKRP*, *MPI*, *MOGS*, *SDR5A3* and *TMEM199*) and another four variants (9%) were detected in damaged DNA binding genes (*EP300*, *MSH6*, *POLD1* and *RECQL4*). These 10 variants, identified in recessive genes, were carried in heterozygosis by 8 patients (Table 2).

Additionally, two variants were found in genes related to coagulation (*PLAU*) and cardiovascular risk (*LDLR*) in two different cases (Table 2). These two cases required intubation and developed severe complications during SARS-COV-2 infection (Supplementary Table 2). The patient carrying the *LDLR* variant was also heterozygous for a variant in *CFI*.

### Network analysis of genes with candidate variants

**Figure 2:**
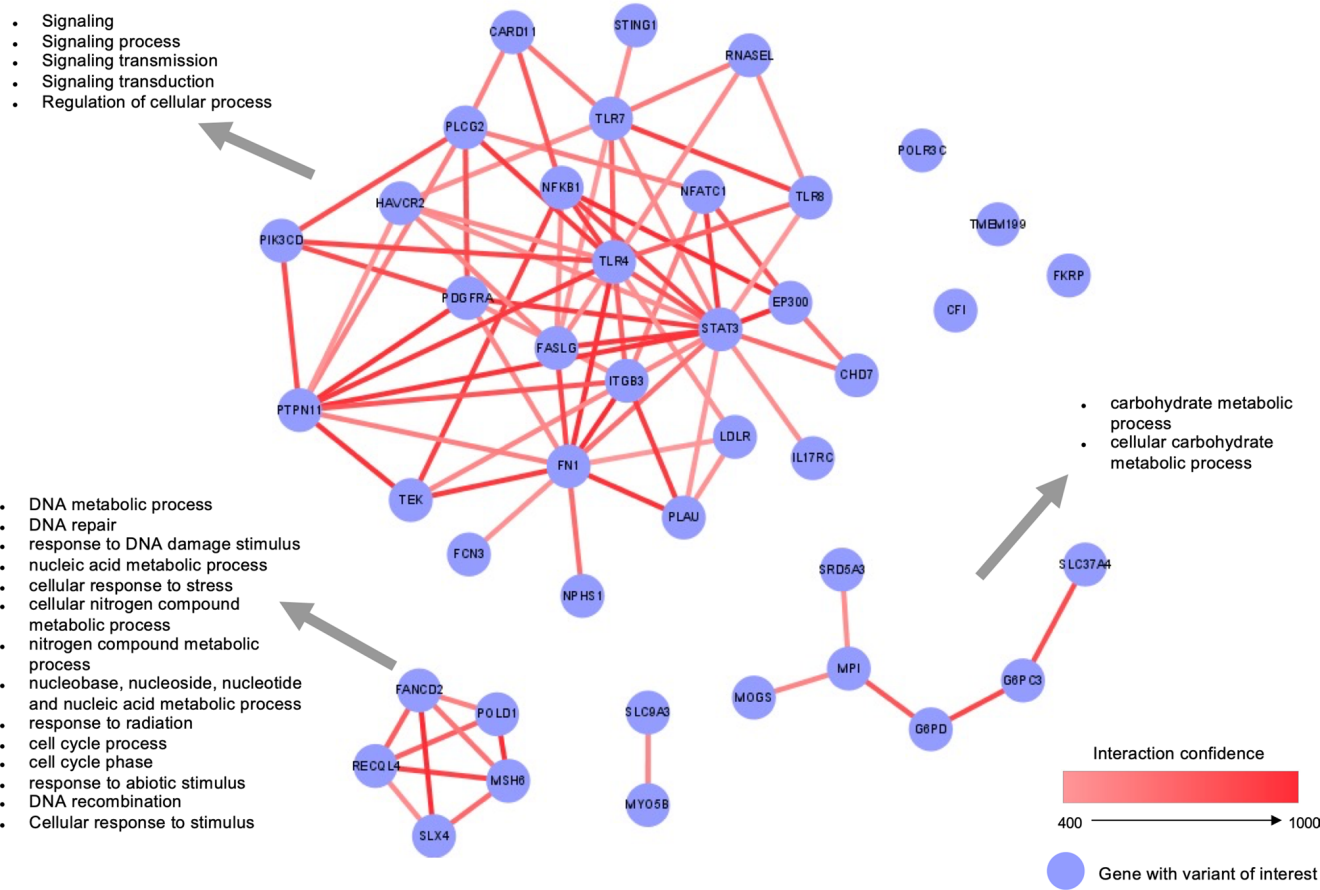
Network analysis of the genes with candidate variants.

A network analysis, including genes with candidate variants (Supplementary Table 3), was performed to detect functional interactions among them. The network (Fig. 2) shows three main components. First component consists of 25 highly interconnected genes, 15 involved in immune response and enriched in cell signaling compared to the rest of the network (Fig. 2). Two additional network components were identified, one composed by 6 genes and enriched in carbohydrate metabolism and a third component with 5 genes enriched in DNA metabolism and repair processes, both compared to the rest of the network.

## Discussion

Understanding inter-individual clinical variability in COVID-19 has important implications for the identification of high-risk patients, clinical decision-making and the development of individualized treatments. In the present study, a group of young and middle-aged patients with very severe COVID-19 were selected for a genetic study, in which 44 different variants of interest have been detected. As expected, most of the detected variants (40%) were encoded by genes directly related to immune response, as the gene panel used in this exploratory study was enriched in immune-related pathways and included additional immune genes than those reported in previous studies^11,13^.

Innate immunity is crucial for early antiviral response; thus, LoF variants in related genes could affect the onset of the immune response and also alter the appropriate clearance of the infection by adaptative response^9,26,27^. In this sense, pathogenic rare variants in 13 candidate genes involved in TLR3- and IRF7-dependent type I IFN pathways showed a higher risk to severe SARS-CoV-2 infection, which could explain up to 3.5% of severe cases^11^. However, these initial findings were not replicated in subsequent studies^12,28^. In our study, we did not find any LoF variant in those 13 genes, but we have detected seven likely pathogenic variants in other genes directly related to immune response (IFN pathways, mainly).We have confirmed the presence of a *TLR7* variant in a male also participating in a recently published study^16^, suffering from very severe COVID-19 and without relevant comorbidities or risk factors at the time of the infection. Therefore, our results support the genetic screening of *TLR7* variants in young men in absence of pre-existing conditions as a preventing biomarker that may help clinical management of this subset of patients. Even more, we have found two variants in other Toll-like receptor (TLRs) genes, *TLR4* (Chr9) and *TLR8* (ChrX), in two males under 50 years of age requiring intubation. Of note, TLRs are crucial in innate response by recognizing pathogen-associated molecular patterns from different microorganisms^29^, being *TLR3, 7* and 8 key sensors of RNA viruses^30^.

Furthermore, nearly 40% of the variants detected in the present study were located in immune response genes, some of them with a high probability of intolerance to heterozygous LoF variation (pLI ≥ 0.9)^31^;thus, a single LoF variant may lead to a severe clinical phenotype due to haploinsufficiency in genes such as *CARD11*, *STAT3* or *NFKB1*(pLI = 1, each). In addition to allelic dosage, subjects carrying the same genotypes can display variable expressivity and additional common or rare genetic variants may modify the penetrance of monogenic variants (polygenic risk)^32^. In this sense, 30% of our patients carried more than one variant of interest. Even more, other well-known COVID-19 risk factors, such as age, comorbidities, or environmental factors may affect monogenic variants penetrance to the final observed phenotype^33^.

Additionally, we found 5 patients (11%) carrying heterozygous variants in genes related to glycosylation defects. Congenital defects of glycosylation (CDG) is a group of rare diseases caused mainly by recessive genes^34^. Clinical manifestations of CDG include neurological, cardiovascular, and hematologic involvement and recurrent infections, among others^35^. An increased risk of thrombotic events and bleeding complications have been related to abnormal glycosylation of coagulation factors^36^ and thrombosis is one of the most important complications of COVID-19. Therefore, patients carrying a defective copy may experience a more severe course of SARS-CoV-2 infection due to the importance of glycosylation in immune response^35^. In contrast, ACE2 is a protein extensively glycosylated and previous studies showed that cellular SARS-CoV-2 entry is reduced by blocking the N-glycan and O-glycan formation^37^. Thus, it is difficult to conclude about the effect of these defective variants on the glycosylation status of the monoallelic carriers and the impact of those variants on SARS-CoV-2 clearance.

Moreover, four variants were detected in damaged DNA binding genes and a cluster including three of these genes (*POLD1*, *MSH6* and *REQL4*), in addition to *SLX4* and *FANCD2*, was detected in the network analysis. There is evidence that senescence is in part caused by accumulated DNA damage^38^ and severity of some pathologies, as COVID-19, has been related to cell senescence, particularly in the elderly^39^. In addition, premature cellular senescence could be induced by viral infections^40^; therefore, COVID-19 patients with pathogenic variants in damaged DNA binding genes may be more likely to develop cellular senescence and severe COVID-19.

Interestingly, one of the candidate variants identified was in the canonical splice site of a key player of the coagulant pathway, *PLAU,* that has been previously related to bleeding disorders, tandem duplication of this gene is related to Quebec platelet disorder (MIM #601709) in a dominant model. Therefore, we could hypothesize that this variant may impair thrombosis resolution, as demonstrated previously in a knocked-out model^41^ and inferred by the critical role of PLAU in the natural thrombus resolution by its fibrinolytic function^42^. Anticoagulant and fibrinolytic gene expression has been found dramatically down-regulated in the lung of COVID-19 patients compared to controls^43^. Thus, COVID-19 patients with loss of functions in the *PLAU* gene may be more likely to develop a thrombotic event. Moreover, we found a variant in the *LDRL* gene, classified as a variant of uncertain significance in relation to familial hypercholesterolemia^44^. Patients with impaired cholesterol metabolism could display a higher risk of COVID-19 severe outcomes due to the intimate relationship of hypercholesterolemia, metabolic syndrome, and heart disease^45^. Therefore, variants predisposing to hypercholesteremia such as *LDLR* pathogenic variants may confer a higher risk of suffering severe COVID-19 disease, even in the absence of other relevant comorbidities^46^.

Our study has several limitations. First, we have a limited sample size. Despite recruiting more than 3500 in the Stop_Coronavirus cohort, the stringent cut-off for age (< 65 years) and outcome (only very severe COVID-19) led us to select those patients displaying an extreme phenotype in our cohort. Second, we have analyzed only the coding region; thus, we could have missed a second pathogenic allele (deep intronic regions or CNVs) in monoallelic patients that could help us explain the COVID-19 outcome. Besides, together with the effect of the detected genetic variants, it is necessary to consider the possible additional effect of pre-existing conditions related to COVID-19 severity in the patients on the outcome.

In conclusion, our descriptive study in very severe COVID-19 patients has reported the presence of rare variants in certain biological pathways such as immune response. Moreover, two additional signaling pathways have been detected including genes involved in carbohydrate metabolisms and DNA repair. Further studies are needed to confirm the ultimate role of the variants described in the present study on COVID-19 severity.

## Patients and methods

### Subjects and clinical data

A case series study was performed by selecting a subgroup of patients (n = 44) from the Spanish STOP_Coronavirus^47^ cohort, which comprises more than 3,500 COVID-19 patients, from 4 hospitals (three from Madrid and one from Murcia). Extreme phenotypes were selected from our STOP_Coronavirus cohort using a similar design to previous case series studies^13^. Inclusion criteria were young and middle-aged patients (age under 65 years) with a confirmatory test of SARS-CoV-2 infection that presented ARDS (survivors or deceased). More information about the Spanish STOP_Coronavirus cohort is provided in Supplementary methodology. Cases were retrospectively and prospectively enrolled from March to May 2020 and followed-up until December 2020. SARS-CoV-2 infection was confirmed by a positive PCR (n = 41) and/or serological test (n = 3, IgG and IgM both positives). COVID-19 patients were recruited from four hospitals in Spain: Hospital Universitario Fundación Jiménez Díaz (HUFJD), Hospital Universitario Infanta Elena (HUIE) and Hospital Universitario 12 de Octubre (H12O) in Madrid, and Hospital Clínico Universitario Virgen de la Arrixaca in Murcia (HVAM).

Clinical data obtained in HUFJD and HUIE were extracted from the patients’ electronic medical records using batch-based complex queries and then reviewed and refined manually by two clinicians and two clinician researchers. At H12O and HVAM, clinical data were manually collected by researchers from electronic medical records. Clinical information included primary demographic data, comorbidities, COVID-19 symptoms, laboratory findings, treatments, related complications from COVID-19, ICU admissions, and outcomes (Supplementary Table 1). Descriptive statistics (mean and SD) were calculated for main clinical and demographic data (Table 1).

This study was approved by the research ethics committees of HUFJD, HVAM and H12O. Wherever was possible, patients provided written or verbal informed consent to participate in this study. Due to the health emergency, the research ethics committees of each center waived the requirement for informed consent for the STOP_Coronavirus cohort. All samples were de-identified (pseudonymized) and clinical data were managed in accordance with national legislation and institutional requirements.

### Ancestry inference

Principal component analysis (PCA) based on the variance-standardized relationship matrix was used to infer the ancestry of each patient and classify them as one of the selected ancestry groups (European, African, admixed American, and East Asian) using a set of 1000 genome samples (phase 3) as a reference population. For PCA, we used previously collected genetic data from our cohort (unpublished) obtained with the Applied Biosystems™ Axiom™ Spain Biobank Array (COL32017 1217, Thermo Fisher Scientific Inc.), which contains 758,740 variants. PCA was performed using Plink software version 1.9^48^.

### Kinship test

To assess kinship, we used previously collected genetic data from our cohort^2^ obtained with the Applied Biosystems™ Axiom™ Spain Biobank Array (COL32017 1217, Thermo Fisher Scientific Inc.), which contains 758,740 variants. Autosomal SNPs (MAF > 5%) were pruned with PLINK^3^ using a window of 1000 markers, a step size of 80 and a r^2^ of 0.1. A subset of 131,937 independent SNPs was used to evaluate kinship (IBD estimation) in PLINK^3^. Only one individual from each pair of individuals with PI_HAT > 0.25 (second-degree relatives) that showed a Z0, Z1, and Z2 coherent pattern (according to theoretically expected values for each relatedness level), was removed.

### Whole exome sequencing analysis

DNA was isolated from EDTA-collected peripheral blood samples using an automated DNA extractor (BioRobot EZ1, QIAGEN GmbH). DNA samples were subjected to library construction using SureSelect Human All Exon V6 (Agilent Technologies, Santa Clara, CA, USA) and sequenced on a Novaseq 6000 instrument (Illumina, San Diego, CA, USA), following the manufacturer’s protocol. Paired-end reads of 2 × 150 bp were generated per sample to provide an on-target coverage of minimum 100 ×, with a total coverage of 12 GB/sample.

For WES analysis we applied an in-house maintained bioinformatics pipeline using bwa v0.7.17^49^ for mapping to the GRCh37/hg19 human genome assembly, gatk v4.2.0 HaplotypeCaller^50^ for single nucleotide variants calling and hard filtering (SNP_filter: QD (Quality of Depth) < 2.0, MQ (Mapping Quality) < 40.0, MQRankSum <  − 12.5, and ReadPosRankSum <  − 8.0, and INDEL_filter: QD < 2.0, and ReadPosRankSum <  − 20.0). Annotations were performed using VEP r103^51^. More details can be seen at the github repository https://github.com/TBLabFJD/VariantCallingFJD and application of the same tool in Romero et al.^52^.

### Single variant analysis

To search for candidate variants involved in the pathophysiology of severe COVID-19, we used a candidate virtual gene panel summarized in Supplementary Table 4. Candidate gene panel included 330 genes mainly involved in type I IFN immunity, primary immunodeficiencies, and genes related to coagulation (panel 1). Moreover, 234 additional genes were selected by using the COVID-19 severity and susceptibility panel published in PanelApp^53^, by selecting only green-labelled genes (panel 2)**.** Besides, other functionally related genes were included by using our GLOWgenes prioritization method (www.glowgenes.org) using the 564 genes from panels 1 and 2 as a seed set. Top 300 prioritized genes were selected and included as panel 3 (Supplementary methodology). Thus, a total of 864 genes (564 candidates and 300 selected by GLOWgenes) were included in the final panel.

The PriorR v.2.1 package (https://github.com/TBLabFJD/PriorR) was used for variant filtering and prioritization**.** Variants were filtered according to a minor allele frequency (MAF) < 0.01 in population databases [the 1000 genomes project^25^, the Exome Aggregation Consortium (*ExAc*)^24^, and the Genome Aggregation Database^23^ (*GnomAD*)]. Synonymous, intronic and non-coding variants were excluded from the analysis. *ClinVar* (ncbi.nlm.nih.gov/clinvar/) and the *Human Gene Mutation Database*^54^ (HGMD) were used to identify variants previously reported as pathogenic and those described as likely benign/benign variants were discarded. The impact of missense variants was assessed using several predictor tools (DANN^55^, FATHMM^56^, GERP++^57^, LRT^58^, M-CAP^59^, CADD^60^, MutationTaster^61^, MutationAssessor^62^, PhyloP^63^, Polyphen2_HDIV^64^, Polyphen2_HVAR^64^, PROVEAN^65^, RadiaISVM^66^, SIFT^67^, SiPhy^68^, among others). Canonical and noncanonical splicing variants were assessed using 5 predictors (MaxEntScan^69^, Human Splicing Finder^70^, Splice Site Finder-like^71^, NNSPLICE^72^, and GeneSplicer^73^) using the Alamut software (Interactive Biosoftware, Rouen, France). The potential pathogenicity of prioritized variants was assessed using the *Varsome* tool^74^ following ACGM criteria^75^.

### Network analysis of genes with candidate variants

Genes carrying at least one of the candidate variants (Supplementary Table 3) were submitted to the STRING database v11.5^76^ and interactions with a STRING combined score ≥ 400 were downloaded as a file (.tsv) in short tabular text output format from the Exports tab. Cytoscape^77^ version 3.4.0 was used for visualization. Clusters were defined as subgraphs with any two nodes (genes) connected to each other by edges, and not connected to other nodes in the graph, this normally called network components and the most extreme version of a cluster. We applied BINGO^78^ Cytoscape app for the enrichment analysis extracting over-represented Gene Ontology (GO) biological processes terms comparing their annotation in every cluster to the rest of the network including genes not grouped in clusters. In the network representation, the STRING combined score, which represents the interaction confidence, is used to characterize edges between genes. Functions enriched for every cluster were selected as having an FDR < 0.05.

## Supplementary Information

Supplementary Legends.

Supplementary Table 1.

Supplementary Table 2.

Supplementary Table 3.

Supplementary Table 4.

Supplementary Information.

## Data Availability

The datasets used and/or analysed during the current study available from the corresponding author on reasonable request.
