# Supplementary material for: Presence of rare potential pathogenic variants in subjects under 65 years old with very severe or fatal COVID-19
Source: Sci Rep. 2022 Jun 20;12:10369. doi: 10.1038/s41598-022-14035-x (PMC9208539; doi:10.1038/s41598-022-14035-x)
Supplement: Supplementary file 1 — Supplementary Legends. [file 41598_2022_14035_MOESM1_ESM.docx]

**SUPPLEMENTARY METHODOLOGY**

**SUPPLEMENTARY TABLES**

**Supplementary Table 1.** Clinical and demographic characteristics of the patients included in present study.

**Supplementary Table 2.**Detailed information about frequency, pathogenic predictors scores and clinical classification of the detected variants.

**Supplementary Table 3.** List of genes with candidate variants included in the network analysis.

**Supplementary Table 4.** List of candidate gene included in the panel.
