# Supplementary material for: Presence of rare potential pathogenic variants in subjects under 65 years old with very severe or fatal COVID-19
Source: Sci Rep. 2022 Jun 20;12:10369. doi: 10.1038/s41598-022-14035-x (PMC9208539; doi:10.1038/s41598-022-14035-x)
Supplement: Supplementary file 6 — Supplementary Information. [file 41598_2022_14035_MOESM6_ESM.pdf]

SUPPLEMENTARY METHODOLOGY

Description of the STOP_Coronavirus cohort

The cohort was collected by a multicentric consortium of four hospitals in Spain: Hospital Universitario Fundación Jiménez Díaz (HUFJD), Hospital Universitario Infanta Elena (HUIE) and Hospital Universitario 12 de Octubre (H12O) in Madrid, and Hospital Clínico Universitario Virgen de la Arrixaca in Murcia (HVAM). The STOP_Coronavirus[1] cohort consists of 3656 patients with a confirmatory test of SARS-CoV-2 infection that were retrospectively and prospectively enrolled from March 2020 to February 2021 and followed up until June 2021. Most of the patients were recruited during the first wave of the pandemic (n=2944, 81%) although the cohort also includes patients from the second (n=625, 17%) and third (n=84, 2%) waves in Spain. Clinical data obtained in HUFJD and HUIE were extracted from the patients' electronic medical records using batch-based complex queries and then reviewed and refined by two clinicians and two clinician researchers independent professionals. At H12O and HVAM, clinical data were manually collected by researchers from electronic medical records. Clinical information included primary demographic data, comorbidities, COVID-19 symptoms, laboratory findings, treatments, related complications from COVID-19, ICU admissions, and outcomes. Fifty-three percent of the patients were males (n=1948) and the median age was 61 years old. The STOP_Coronavirus cohort is included in the Spanish COalition to Unlock Research on host GEnetics on COVID-19 (SCOURGE)[2].

Subjects and clinical data

A case series study was performed by selecting a subgroup of patients from the Spanish STOP_Coronavirus cohort described above. Patients included in the present study meet the following inclusion criteria:

- Patients of the first wave (March to May 2020)
- Confirmatory test of SARS-CoV-2 infection
- Severe distress respiratory syndrome aged under 65 years

A total of 44 patients were included following these criteria. Clinical data obtained in HUFJD and HUIE were extracted from the patients' electronic medical records using batch-based complex queries and then reviewed and refined manually by two clinicians (JRH and BA) and two clinician researchers (MC and RLR) independent professionals. At H12O and HVAM, clinical data were manually collected by researchers from electronic medical records. Clinical information included primary demographic data, comorbidities, COVID-19 symptoms, laboratory findings, treatments, related

complications from COVID-19, ICU admissions, and outcomes (Supplementary Table 1). Descriptive statistics (mean and SD) were calculated for main clinical and demographic data (Table 1).

Gene panel design

The gene panel used for prioritization includes genes involved in immune response (mainly type I IFN immunity), primary immunodeficiencies, and genes related to coagulation and fibrinolysis. This was denoted as panel 1 and included a total of 330 genes. Additionally, 234 genes were selected by using the "COVID-19 severity and susceptibility panel" published in PanelApp[4], by selecting only green-labeled genes and denoted as panel 2. Finally, a third panel (panel 3) was defined using the GLOWgenes candidate genes prioritization method (glowgenes.org). GLOWgenes is a network-based algorithm that performs gene annotation expansion using as input a list of genes, in our case, the genes selected for the panel, a random-walk with restart propagation model (RWWR) as annotation model and 33 gene-gene functional networks of different entity. The propagation of the phenotype annotation over every network is evaluated and the integration of all annotations was performed in a single ranking that sorts the whole genome according to the functional similarity to the input list of genes. Top 300 prioritized genes were selected and included in the panel. GLOWgenes is currently in its first version and next releases will be available at the github repository: https://github.com/TBLabFJD/GLOWgenes**.** Thus, a total of 864 genes (564 candidates and 300 selected by GLOWgenes) were included in the final panel (Supplemantary Table 4).

Panel 1: *ABCB1, ABO, ACE, ACE2, ACP5, ADA, ADAR, AICDA, AIRE, ALG1, ALG11, ALG12, ALG14, ALG3, ALG6, ALG8, ALG9, AP3B1, APOL1, ATP6V0A2, B2M, B3GALNT2, B3GALT6, B3GAT3, B3GLCT, B4GALNT1, B4GALT1, B4GALT7, BCL10, BTK, C1QA, C1QB, C1QC, C1R, C1S, C3, CARD11, CARD9, CASP10, CASP8, CCDC115, CCHCR1, CCL2, CCL5, CCL8, CCR2, CCR5, CCR9, CD19, CD209, CD247, CD27, CD3D, CD3E, CD3G, CD40, CD46, CD55, CD81, CFH, CFHR1, CFHR5, CFI, CHST14, CHST3, CHST6, CHSY1, COG1, COG4, COG5, COG6, COG7, COG8, CORO1A, CR2, CSF2, CSGALNACT1, CTLA4, CXCL10, CXCL8, CXCR6, CYBB, DNASE1L3, DOCK8, DOLK, DPAGT1, DPM1, DPM2, DPM3, DPP9, EIF2AK2, EOGT, EXT1, EXT2, FADD, FANCD2, FAS, FASLG, FCGR2A, FCGR2B, FCN3, FKRP, FKTN, FOXP3, FUK, FUT8, FYCO1, G6PC3, G6PD, GALNT2, GALNT3, GATA2, GFPT1, GMPPA, GMPPB, GNE, GORAB, GSPC3, HAVCR2, HBEGF, HLA-DPB1, HLA-G, HNF1A, ICAM1, ICAM3, ICAM5, ICOS, IF2NB1, IFIH1, IFIT1, IFIT2, IFITM1, IFITM3, IFNA1, IFNA2, IFNAR1, IFNAR2, IFNG, IFNGR1, IFNGR2, IFNL1, IFNL2,*

IFNL3, IFNL4, IFNLR1, IGF1, IKBKB, IKBKG, IKZF1, IKZF3, IL10, IL10RB, IL12A, IL12B, IL12RB1, IL17A, IL17B, IL17F, IL17RA, IL17RC, IL21R, IL22, IL2RA, IL2RG, IL6, IL6R, IRAK1, IRAK4, IRF1, IRF3, IRF4, IRF7, IRF8, IRF9, ISPD, ITK, JAK1, JAK2, KDR, LARGE1, LCK, LDLR, LEP, LRBA, LTA, LZTFL1., MALT1, MAN1B1, MAN2B1, MASP2, MAVS, MBL2, MCM4, MGAT2, MPDU1, MPI, MS4A1, MUC5B, MX1, MYD88, MYH9, MYO5B, NCF2, NEUROG3, NFKB1, NFKB2, NGLY1, NOD2, NOTCH4, NPHS1, NR0B1, OAS1, OAS2, OAS3, OASL, ORAI1, PDCD1, PEPD, PGAP2, PGAP3, PGF, PGM1, PIAS1, PIAS2, PIGA, PIGL, PIGM, PIGN, PIGO, PIGS, PIGT, PIGV, PIGW, PIK3CD, PIK3R1, PLAAT4, PLAU, PLAUR, PLCG2, PLG, PMM2, POMGNT1, POMGNT2, POMK, POMT1, POMT2, PRKDC, PRKRA, PSMB8, PTPN22, PTPRC, RAG2, RASGRP1, REL, RFT1, RNASEH2B, RNASEH2C, RNASEL, SAMHD1, SBDS, SEC23B, SERPINE1, SH2D1A, SIRT1, SLC26A3, SLC35A1, SLC35A2, SLC35D1, SLC37A4, SLC39A8, SLC6A20, SLC9A3, SLX4, SOCS1, SOCS2, SOCS3, SPINT2, SRD5A3, SSR3, SSR4, ST3GAL3, ST3GAL5, STAT1, STAT2, STAT3, STAT4, STIM1, STK4, STT3A, TBK1, TCF3, TEK, TFRC, TGFBI, THBD, THBS1, THSD7A, TICAM1, TIE1, TLR3, TLR4, TLR5, TLR7, TLR8, TMEM165, TMEM173, TMEM199, TMEM5, TMPRSS2, TNFRSF13B, TNFRSF13C, TNFSF4, TRAF3, TRAF6, TRAPPC11, TRIM21, TTC37, TUSC3, TYK2, UNC119, UNC93B1, UNG, VEGFA, VEGFB, VEGFD, WAS, XCR1, XKR6, XYLT1, XYLT2, ZAP70

Panel 2: *ACD, ACTB, ADA2, ADAM17, AIM2, AK2, ALPI, AP1S3, AP3D1, ARHGEF1, ARPC1B, ATM, ATP6AP1, BACH2, BCL11B, BLM, BRIP1, C17orf62, C2, C5, C6, C7, C8A, C8B, C8G, C9, CARD14, CARMIL2, CASP3, CCBE1, CD244, CD59, CDC42, CDCA7, CEBPE, CFD, CFHR4, CFP, CFTR, CHD7, CIB1, CLCN7, CLPB, COPA, CTC1, CTPS1, CTSC, DAG1, DBR1, DCLRE1B, DCLRE1C, DEF6, DICER1, DKC1, DNAJC21, DNASE2, DNMT3B, EFL1, EPG5, ERBIN, ERCC4, ERCC6L2, EXTL3, F12, FAAP24, FANCB, FANCC, FANCE, FANCF, FANCG, FANCI, FANCL, FANCM, FAT4, FCHO1, FERMT1, FERMT3, FOXN1, FPR1, FPR2, GATA1, GFI1, GINS1, HAVCR1, HAX1, HDAC6, HELLS, HMOX1, HPS1, HPS4, HPS6, HTRA2, HYOU1, IGHM, IGKC, IGLL1, IL18BP, IL36RN, INO80, IRF2BP2, ITCH, ITGAV, ITGB3, ITPKC, JAGN1, KDM6A, KIAA0319L, KLF2, KMT2A, KMT2D, LAMTOR2, LIG1, LIG4, LPIN2, LYST, MAD2L2, MAGT1, MAP3K14, MEFV, MKL1, MOGS, MPO, MSH6, MSN, MTHFD1, MVK, MYSM1, NBAS, NBN, NCSTN, NFAT5, NFE2L2, NHEJ1, NHP2, NLRP1, NLRP12, NOP10, NOS2, NSMCE3, OSTM1, OTULIN, PALB2, PARN, PAX1, PGM3, PIK3CG, PLEKHM1, PMS2, PNP, POLA1, POLD1, POLD2, POLE, POLE2, POLR3A, POLR3C, POLR3F, POMP, PSEN1, PSENEN, PSMG2, PSTPIP1, RAB27A, RAC2, RAD51, RAD51C, RANBP2, RBCK1, RECQL4, RFWD3, RFX5, RFXANK, RFXAP, RHOH, RMRP, RNF168, RNF31, RNU4ATAC, RORC, RPSA, RTEL1, SAMD9, SAMD9L, SEC61A1, SEMA3E, SGPL1, SH3KBP1, SKIV2L, SLC29A3, SLC35C1, SLC39A7, SLC46A1, SLC7A7, SMARCAL1, SMARCD2, SNORA31, SNX10, SP110, SPINK5, SPPL2A, SRP54, SRP72, STN1, STX11, STXBP2, TAP2, TAPBP, TAZ, TBX1, TCIRG1, TCN2, TERC, TERT, TGFBR1, TGFBR2, TINF2, TMC6, TMC8, TNFRSF11A, TNFRSF9, TOP2B, TPP2, TRAC, TRIM22, TRNT1, TTC7A, UBE2T, UNC13D, USB1, VPS13B, VPS45, WDR1, WRAP53, XRCC2, ZBTB24, ZNF341*

Panel 3 (GLOWgenes): *AGT, AKT1, APOA1, BATF, BCL2, BCL3, BCL6, BIRC2, BIRC3, BLK, BLNK, BST2, C4A, C4B, CASP1, CASP4, CBL, CCL11, CCL17, CCL18, CCL19, CCL20, CCL21, CCL22, CCL3, CCL4, CCL7, CCR1, CCR3, CCR6, CCR7, CCRL2, CD14, CD1C, CD1D, CD2, CD274, CD28, CD33, CD38, CD4, CD40LG, CD44, CD5, CD68, CD69, CD70, CD74, CD79A, CD79B, CD80, CD83, CD86, CD8A, CD8B, CEBPA, CEBPB, CFB, CFHR2, CFHR3, CFLAR, CHUK, CIITA, CISH, CLEC7A, CR1, CRP, CSF1, CSF1R, CSF2RA, CSF2RB, CSF3, CSF3R, CXCL1, CXCL11, CXCL13, CXCL16, CXCL2, CXCL9, CXCR2, CXCR3, CXCR4, CXCR5, CYBA, DDX58, DHX58, DOCK2, DPP4, EBI3, EGFR, ELANE, EP300, ERBB2, ETS1, FANCA, FCGR1A, FCGR3A, FCN1, FLT1, FLT3, FN1, FOS, FYB1, FYN, GATA3, GBP1, GRB2, GZMB, HCK, HIF1A, HLA-A, HLA-B, HLA-C, HLA-DRB1, HLA-E, HRG, ICOSLG, IDO1, IFI16, IFI35, IFI44, IFI6, IFIT3, IFITM2, IFNA10, IFNA13, IFNA14, IFNA16, IFNA17, IFNA21, IFNA4, IFNA5, IFNA6, IFNA7, IFNA8, IFNE, IFNK, IFNW1, IKBKE, IL10RA, IL12RB2, IL13, IL13RA1, IL15, IL15RA, IL17C, IL17RB, IL17RE, IL18, IL19, IL1A, IL1B, IL1R1, IL1RN, IL2, IL20RB, IL21, IL22RA1, IL22RA2, IL23A, IL23R, IL24, IL25, IL26, IL27, IL27RA, IL2RB, IL3, IL3RA, IL4, IL4R, IL5, IL6ST, IL7, IL7R, IL9, IL9R, INPP5D, IRAK2, IRAK3, IRF2, IRF5, ISG15, ITGAL, ITGAM, ITGAX, ITGB2, JAK3, JUN, KIT, KITLG, LAT, LCP2, LGALS9, LRCH1, LTB, LY9, LY96, LYN, MAP3K7, MAP3K8, MAPK1, MMP9, MPL, MYC, NCF1, NCF4, NFATC1, NFATC2, NFKBIA, NLRC4, NLRC5, NLRP3, NOD1, NRP1, OSM, PDCD1LG2, PDGFRA, PDGFRB, PELI1, PF4, PIK3CA, PLAT, PRDM1, PRF1, PRKCD, PRKCQ, PTAFR, PTGS2, PTPN11, PTPN2, PTPN6, PTX3, PYCARD, RAG1, RELA, RELB, RIPK1, RIPK2, RIPK3, RNASEH2A, RSAD2, RUNX3, SELL, SELP, SERPING1, SHC1, SIGIRR, SLAMF1, SMAD4, SP1, SPI1, SPIB, SRC, STAT5A, STAT5B, STAT6, SYK, TANK, TAP1, TBX21, TGFB1, TICAM2, TIRAP, TLR1, TLR10, TLR2, TLR6, TLR9, TNF, TNFAIP3, TNFRSF10A, TNFRSF10B, TNFRSF1A, TNFRSF1B, TNFRSF4, TNFSF10, TNFSF11, TNFSF12, TNFSF13, TNFSF13B, TNFSF14, TNIP1, TP53, TRADD, TRAF1, TRAF2, TRAF3IP2, TRAF5, TRAT1, TRIM25, TSLP, TYROBP, USP18, VAV1, VCAM1, WIPF1, XCL1, XIAP, ZBP1*

Enrichment analysis of the complete gene panel

An enrichment analysis was performed using the Enrichr tool[5]where the input was the complete list of genes in the panel. Top10 KEGG pathways and top10 Gene Ontology terms for biological processes are shown.

Enrichment analysis of KEGG pathways in the complete gene panel

Total significant KEGG pathways (q-value < 0.05) = 157

Top 10 KEGG pathways over-represented

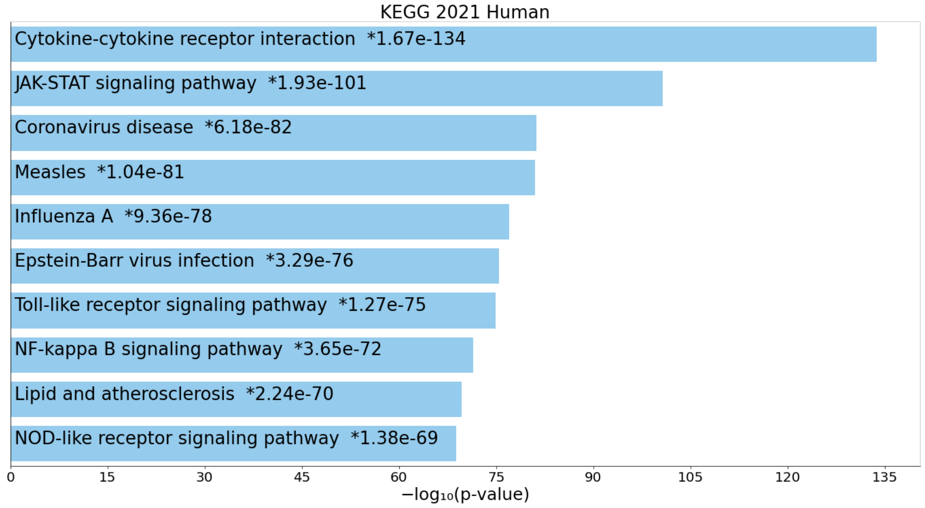

Enrichment analysis of Gene Ontology (biological processes) in the complete gene panel

Total significant GO terms (q-value < 0.05) = 1522

Top 10 GO BP terms over-represented

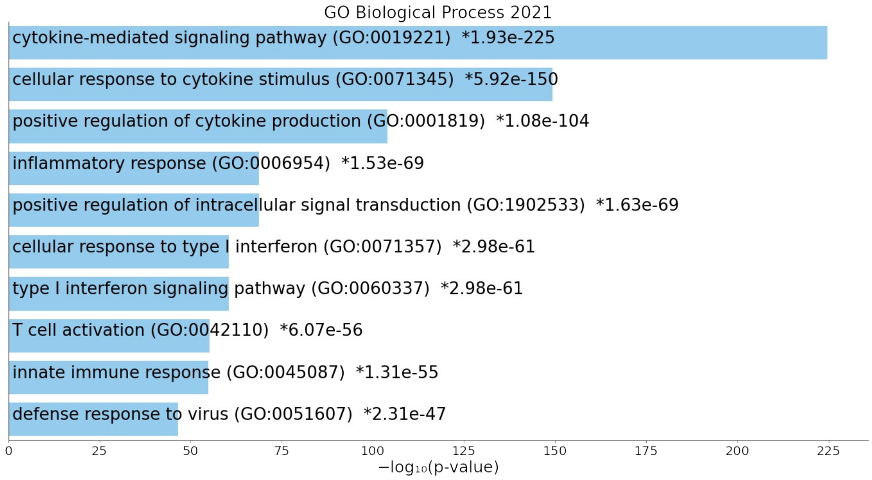

References

1. Lopez-Rodriguez, R. *et al.* Androgen receptor polyQ alleles and COVID-19 severity in men: a replication study. *medRvix* (2022) doi:10.1101/2022.03.25.22271678.
2. Carracedo, Á. & (SCOURGE), S. Co. to U. R. on host Ge. on C.-19. A genome-wide association study of COVID-19 related hospitalization in Spain reveals genetic disparities among sexes. *medRxiv* 2021.11.24.21266741 (2021) doi:10.1101/2021.11.24.21266741.
3. Chang, C. C. *et al.* Second-generation PLINK: rising to the challenge of larger and richer datasets. *Gigascience* **4**, 7 (2015).
4. Martin, A. R. *et al.* PanelApp crowdsources expert knowledge to establish consensus diagnostic gene panels. *Nat. Genet.* **51**, 1560–1565 (2019).
5. Chen, E. Y. *et al.* Enrichr: interactive and collaborative HTML5 gene list enrichment analysis tool. *BMC Bioinformatics* **14**, 128 (2013).
